# Cilostazol-based dual antiplatelet treatment in ischemic stroke or transient ischemic attack patients with asymptomatic carotid artery disease: a propensity score matching analysis

**DOI:** 10.3389/fneur.2024.1362124

**Published:** 2024-02-15

**Authors:** Thon Thiraworawong, Chadawan Pathonsmith

**Affiliations:** ^1^Division of Vascular Neurology, Department of Neurology, Neurological Institute of Thailand, Bangkok, Thailand; ^2^Division of Geriatric Medicine, Faculty of Medicine, Vajira Hospital, Navamindradhiraj University, Bangkok, Thailand

**Keywords:** dual antiplatelet, ischemic stroke, asymptomatic, cilostazol, carotid stenosis

## Abstract

**Background:**

The optimal treatment for asymptomatic atherosclerotic carotid artery disease remains controversial. Data on the efficacy of antiplatelet agents and stroke outcomes are limited. This study aimed to examine the efficacy and safety of cilostazol-based dual antiplatelet therapy in patients with ischemic stroke or transient ischemic attack and asymptomatic carotid artery disease.

**Methods:**

This retrospective cohort study was conducted in a tertiary-care setting and included baseline characteristics and clinical outcomes of participants. The study included patients who had experienced first-ever ischemic stroke or transient ischemic attack and asymptomatic atherosclerotic carotid artery stenosis, with a minimum follow-up period of 1 year. Asymptomatic carotid artery stenosis refers to stenosis in patients without neurological symptoms referable to the carotid arteries. Propensity scores were estimated using a logistic regression model based on participants’ baseline characteristics. The efficacy outcome was the composite outcome of recurrent ischemic events and vascular-related death in patients with ischemic stroke or transient ischemic attack and asymptomatic carotid artery stenosis. The safety outcome was the occurrence of hemorrhagic complications such as intracranial hemorrhages or extracranial hemorrhages. The effectiveness of dual therapy compared to monotherapy was evaluated at various time points following the initiation of antiplatelet treatment.

**Results:**

This study included 516 patients with a 1-year follow-up period. At 1 year, composite events occurred in 10 (6.3%) patients in the dual antiplatelet group compared with 12 (7.6%) in the single antiplatelet group (HR, 0.74; 95% CI, 0.61–0.90; *p* = 0.024). Extracranial hemorrhage occurred in 12 (7.6%) patients in the dual antiplatelet group compared with nine (5.7%) in the single antiplatelet group (HR, 1.35; 95% CI, 1.13–1.48; *p* = 0.017). No intracranial hemorrhages were observed in this cohort.

**Conclusion:**

Patients with asymptomatic carotid artery stenosis who received cilostazol-based dual antiplatelet therapy had a lower risk of composite events but a higher risk of minor extracranial hemorrhage than those who received a single antiplatelet agent.

## Introduction

Ischemic stroke can result from carotid artery disease. The treatment approach for symptomatic carotid artery stenosis is well established; however, the optimal approach for asymptomatic carotid artery stenosis remains controversial. Asymptomatic carotid artery stenosis refers to stenosis in persons without a history of ischemic stroke, transient ischemic attack, or other neurologic symptoms referable to the carotid arteries. Regarding diagnosing carotid artery stenosis, one usually uses the least invasive and inexpensive test, such as carotid doppler ultrasonography. As a confirmatory test, some form of contrast examination such as digital subtraction angiography, magnetic resonance angiography or computed tomographic angiography is required to confirm the diagnosis. Current treatment guidelines recommend medical therapy and carotid revascularization, including carotid endarterectomy and stenting, for asymptomatic carotid artery stenosis with a high risk of progression. The limitations of previous studies have resulted in different treatment guidelines for patients with asymptomatic carotid artery stenosis. Therefore, early evaluation and appropriate treatment of asymptomatic carotid artery stenosis are crucial. The best medical treatment has evolved from its loose definition in asymptomatic carotid artery trials and now encompasses different antiplatelet regimens (1). Dual antiplatelet therapy has demonstrated efficacy in reducing the recurrence of cerebral ischemia (2, 3). Evidence regarding the association between outcomes and type of dual antiplatelet regimen in patients with asymptomatic carotid artery disease is limited. Landmark trials of dual antiplatelet therapy in certain populations, including patients with asymptomatic carotid artery stenosis, have not been specifically evaluated. Additionally, the outcomes from randomized controlled trials may not accurately reflect real-world clinical practice. Data on the efficacy of dual antiplatelet efficacy are scarce. A large multicenter real-life study is currently at the ending point and preliminary results have shown substantial discrepancies from randomized controlled trials (4). Therefore, this study aimed to examine the efficacy and safety of cilostazol-based dual antiplatelet therapy in patients with ischemic stroke or transient ischemic attack and asymptomatic carotid artery disease.

## Materials and methods

### Study design

This retrospective cohort study was conducted between January 2018 and September 2022 at the Neurological Institute of Thailand, a tertiary care and referral center for neurological disorders.

### Study participants

The participants in this study were individuals who had experienced their first-ever transient ischemic attack or acute ischemic stroke and asymptomatic atherosclerotic carotid artery stenosis, having at least 1 year of follow-up, were included in this study. A vascular neurologist (T. T.), and a geriatrician (C.P.) evaluated the medical records of these patients. Transient ischemic attack or ischemic stroke patients were adjudicated by consensus between the two investigators, and any discrepancies were excluded. Only patients whose eligibility was confirmed by both investigators were included in this study.

Patients were excluded from this study if they (1) had carotid artery disease from other mechanisms such as radiation-related carotid stenosis/occlusion, Takayasu disease, fibromuscular dysplasia; (2) received vitamin K oral antagonist or direct oral anticoagulant; (3) had underwent carotid endarterectomy or carotid artery stenting for asymptomatic carotid artery stenosis; (4) had moderate or severe intracranial atherosclerosis; (5) had cardiac arrhythmia or congestive heart failure.

**Figure 1:**
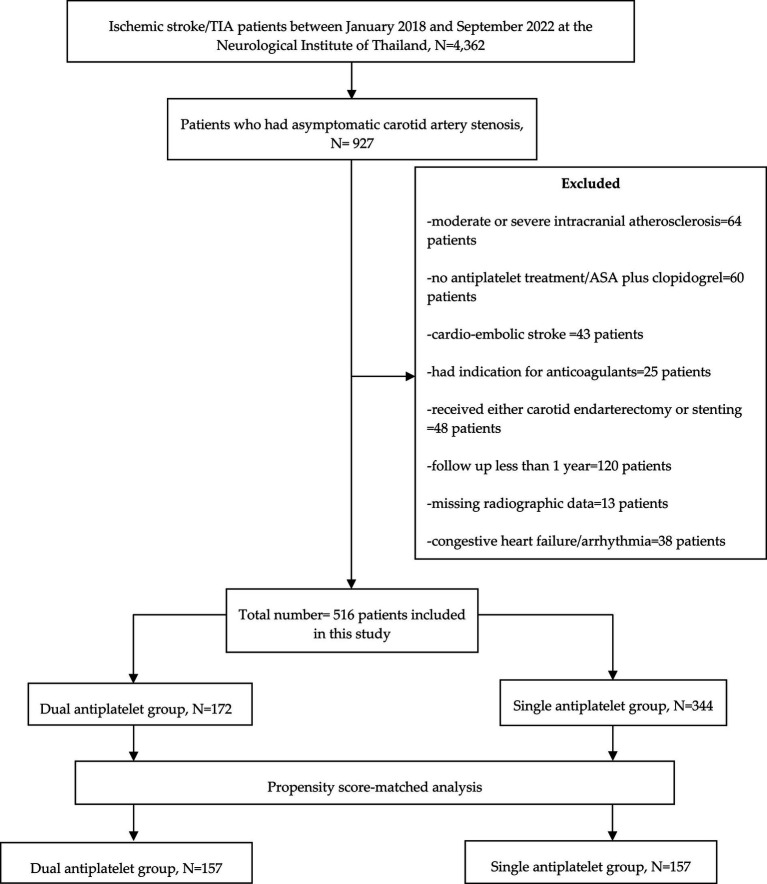
Flow diagram of the study.

Study participants were categorized into two groups: (1) patients who received cilostazol-based dual antiplatelet therapy and (2) those who received single antiplatelet therapy (Figure 1).

### Data collection and definitions

The patients’ baseline characteristics included sex, age, baseline National Institutes of Health Stroke Scale (NIHSS), baseline ABCD^2^ score (the risk of stroke on the basis of age, blood pressure, clinical features, duration of transient ischemic attack, and presence or absence of diabetes), antiplatelet agent type, treatment duration, underlying disease, smoking, low-density lipoprotein cholesterol level (LDL-C), HbA1c level, radiographic data of carotid artery disease, medication non-adherence, and the Fazekas scale. We excluded patients with incomplete or incorrect data for the following variables: age, NIHSS, ABCD^2^, HbA1C level, LDL-C level, clopidogrel loading dose, and time to dual antiplatelet therapy.

Cilostazol-based dual antiplatelet therapy was defined as a combination of 200 mg/day of cilostazol and either aspirin 81–100 mg per day or clopidogrel (75 mg/day).

Single antiplatelet therapy was defined as aspirin 81–325 mg per day or clopidogrel 75 mg/day.

Medication adherence was monitored through electronic prescription refills, with non-adherence defined as the proportion of patients with a refill lag >1 month during follow-up over 1 year.

Achieved target blood pressure was defined as the proportion of patients with hypertension whose systolic blood pressure measured at each visit was <140 mmHg (<130 mmHg if diabetic) for >80% of the follow-up visits within 1 year.

Achieved target LDL-C level was defined as the proportion of patients with hypercholesterolemia with LDL-C levels measured at each visit <1.8 mmol/L for >80% of the follow-up visits within 1 year.

Achieved target HbA1c was defined as the proportion of patients with diabetes mellitus with HbA1c levels measured at each visit of <53 mmol/mol for >80% of the follow-up visits within 1 year.

Smoking cessation was defined as the proportion of smokers who discontinued tobacco smoking within 6 months of the onset of ischemic stroke/transient ischemic attack.

Asymptomatic carotid artery stenosis refers to stenosis in first-ever transient ischemic attack or acute ischemic stroke patients without neurological symptoms referable to the carotid arteries. Asymptomatic carotid artery stenosis was defined as the presence of atherosclerotic narrowing of the proximal internal carotid artery by ≥50% at the level of bifurcation.

Ischemic events were classified as recurrent ischemic stroke or transient ischemic attacks. Recurrent ischemic stroke was defined as the first episode of neurological deficit persisting for over 24 h, localized to previous asymptomatic carotid artery stenosis. Recurrent transient ischemic attack was defined as a focal and localizable (such as hemispheric neurological deficit) transient ischemic attack or monocular blindness persisting for less than 24 h, localized to a previous asymptomatic carotid artery stenosis.

Carotid artery stenosis can be diagnosed using either gadolinium-enhanced magnetic resonance angiography or contrast-enhanced computed tomographic angiography and measured using the North American Symptomatic Carotid Endarterectomy Trial (NASCET) (5) criteria.

Hemorrhagic complications were classified as major or minor. Major hemorrhage was defined as (1) life-threatening hemorrhage with or without any blood component transfusion, (2) hemorrhage requiring any blood component transfusion, and (3) hemorrhage in visceral/vital organs, such as intra-orbital hemorrhage, intracranial hemorrhage, or intraspinal hemorrhage. Minor hemorrhage was defined as any hemorrhagic event not meeting the criteria for major hemorrhage.

Death was defined as vascular-related death, such as myocardial infarction or stroke-related death.

Number of new ischemic lesions, which were defined as any new ischemic lesions apart from the index lesions on follow-up fluid attenuation inversion recovery using slice-to-slice comparison with the baseline diffusion-weighted imaging and fluid attenuation inversion recovery.

### Efficacy outcomes

The primary efficacy outcome of this study was the first episode of composite recurrent ischemic events and vascular-related death. The secondary efficacy outcomes included the first occurrence of ischemic event recurrence, vascular-related death, and number of new ischemic lesions.

### Safety outcomes

The primary safety outcome of this study was the occurrence of hemorrhagic complications such as intracranial hemorrhages or extracranial hemorrhages. Other secondary safety outcome was the occurrence of adverse events.

### Statistical analyses

Propensity scores were estimated using a logistic regression model based on the study participants’ baseline characteristics. We matched the dual antiplatelet treatment group with the single antiplatelet treatment group in a 1:1 ratio using the nearest-neighbor matching method.

We calculated that a sample size of 516 patients would provide 80% power and thereby avoid a type 2 error to detect a 64% risk reduction in the dual antiplatelet treatment group, assuming an 11% event rate of the composite endpoint in the single antiplatelet group (6–8). This study’s sample size calculation formula was derived from a textbook (9). Categorical variables were reported as numbers (%), while continuous variables were reported as mean ± SD or median and interquartile range (IQR) as appropriate. Survival analysis for composite outcomes was estimated using Kaplan–Meier survival curves and compared using Cox regression analysis. Hazard ratios with 95% confidence intervals (CI) are reported. If multiple events of similar type occurred, the time to the first event was used in the model. A value of *p* less than 0.05 was considered significant. Statistical analyses were performed using SPSS for Windows (version 22.0; IBM Corp., Armonk, NY).

## Results

### Baseline characteristics of the study participants

Between January 2018 and September 2022, 4,362 transient ischemic attack or acute ischemic stroke patients were admitted to the stroke unit. 927 patients who had experienced their first-ever transient ischemic attack or acute ischemic stroke and asymptomatic atherosclerotic carotid artery disease were enrolled. This study excluded 64 (6.9%) patients who had moderate or severe intracranial atherosclerosis, 60 (6.5%) patients who had no antiplatelet treatment or received combination of aspirin plus clopidogrel, 43 (4.6%) patients who had cardio-embolic stroke, 25 (2.7%) patients who had indication for anticoagulants, 48 (5.2%) patients who had underwent either carotid endarterectomy or stenting, 120 (12.9%) patients who had follow up less than 1 year, and 38 (4.1%) patients who had congestive heart failure or cardiac arrhythmia. Nevertheless, none of the continuous variables were absent in the database. However, 13 patients were excluded due to the absence of radiographic data (Figure 1).

This single-center, tertiary care setting included 516 patients (Figure 1) with asymptomatic carotid artery stenosis (308 men and 208 women), with a median age of 64 years (Table 1). The baseline characteristics of the study participants are summarized in Table 1. Before propensity score matching, the proportion of patients with coronary artery disease was higher in the dual antiplatelet group. After propensity score matching, all imbalanced baseline characteristics between the study groups were well-balanced, eliminating significant differences.

**Table 1:** Demographic data of the study participants.

Characteristics	Overall cohort (*N* = 516)			Propensity score-matched cohort (*N* = 314)		
	Dual antiplatelet (*N* = 172)	Single antiplatelet (*N* = 344)	*p* value	Dual antiplatelet (*N* = 157)	Single antiplatelet (*N* = 157)	*p* value
Male gender (%)	96 (55.8)	212 (61.6)	0.525	89 (56.7)	92 (58.6)	0.764
Age, years (IQR)	63 (57–71)	65 (56–72)	0.458	64 (57–70)	65 (57–71)	0.529
Body mass index, kg/m2 (IQR)	22.2 (18.2–25.5)	23.1 (17.9–24.4)	0.414	22.1 (19.8–24.4)	22.3 (19.9–24.1)	0.357
Transient ischemic attack/ischemic stroke patients (%)	34 (19.8)/ 138 (80.2)	70 (20.3)/ 274 (79.7)	0.211	28 (17.8)/ 129 (82.2)	27 (17.2)/ 130 (82.8)	0.482
Baseline NIHSS (IQR)	6 (3–8)	7 (3–9)	0.326	6 (3–8)	6 (3–8)	0.544
Baseline ABCD2 score (IQR)	4 (3–6)	4 (3–5)	0.437	4 (3–5)	4 (3–5)	0.546
Hypertension (%)/Achieved target blood pressure (%)	134 (77.9)/ 101 (75.4)	271 (78.8)/ 264 (76.7)	0.534/ 0.433	118 (75.1)/ 88 (74.6)	121 (77.1)/ 89 (73.6)	0.612/ 0.531
Diabetes mellitus (%)/Achieved target HbA1c (%)	106 (61.6)/ 88 (83.0)	202 (58.7)/ 171 (85.5)	0.457/ 0.492	94 (59.9)/ 78 (83.7)	97 (61.8)/ 79 (82.4)	0.358/ 0.515
Hypercholesterolemia (%)/Achieved target LDL-C (%)	115 (66.7)/ 87 (75.7)	216 (62.8)/ 174 (80.5)	0.638/ 0.319	101 (64.3)/ 75 (74.3)	105 (66.9)/ 76 (72.4)	0.732/ 0.431
Medication non-adherence (%)	9 (5.2)	16 (4.7)	0.267	6 (3.8)	7 (4.6)	0.381
Chronic kidney disease (%)	15 (8.7)	41 (11.9)	0.535	14 (8.9)	12 (7.6)	0.478
Coronary artery disease (%)	55 (31.9)	72 (20.9)	0.078	53 (33.8)	56 (35.7)	0.347
Smoking (%)/Smoking cessation (%)	106 (61.6)/ 86 (81.1)	233 (67.7)/ 192 (82.4)	0.554/ 0.659	103 (65.7)/ 84 (81.6)	105 (66.9)/ 86 (82.0)	0.718/ 0.594
HbA1c, mmol/mol (IQR)	52 (48–59)	52 (47–58)	0.432	52 (48–59)	52 (48–57)	0.851
LDL-C, mmol/L (IQR)	1.7 (1.5–2.7)	1.7 (1.5–2.8)	0.358	1.7 (1.6–2.5)	1.7 (1.5–2.6)	0.544
Degree of stenosis >70% (%)	41 (23.8)	91 (26.4)	0.445	39 (24.8)	42 (26.7)	0.312
Fazekas scale (IQR) Periventricular white matter Deep white matter	1 (0–2) 1 (0–2)	1 (0–2) 1 (0–2)	0.388	1 (0–2) 1 (0–2)	1 (0–2) 1 (0–2)	0.459

Table 2 shows the types of antiplatelet treatments used by study participants. The median duration of the dual antiplatelet therapy was 6 months. In this study, 97(56.4%) patients received cilostazol plus clopidogrel, and 75(43.6%) received cilostazol plus aspirin.

**Table 2:** Types of antiplatelet treatments used by study participants.

Characteristics	Overall cohort (*N* = 516)		Propensity score-matched cohort (*N* = 314)	
	Dual antiplatelet (*N* = 172)	Single antiplatelet (*N* = 344)	Dual antiplatelet (*N* = 157)	Single antiplatelet (*N* = 157)
Type of antiplatelet (%)				
Cilostazol plus Clopidogrel	97 (56.4)	–	87 (55.4)	–
Cilostazol plus Aspirin	75 (43.6)	–	70 (44.6)	–
Clopidogrel alone	–	203 (59.0)	–	90 (57.3)
Aspirin alone	–	141 (41.0)	–	67 (42.7)
Clopidogrel 300 mg loading dose (%)	88 (90.7)	188 (92.6)	81 (93.1)	83 (92.2)
Time to start dual antiplatelet, after onset of TIA/ischemic stroke, days (IQR)	2 (1–5)	–	2 (1–5)	–
Duration of dual antiplatelet therapy, months (IQR)	6 (5–7)	–	6 (5–7)	–

### Six month outcomes of dual antiplatelet treatment in propensity score-matched cohort

Table 3 shows the 6-month outcomes of dual antiplatelet therapy in the propensity score-matched cohort. Composite events occurred in six (3.8%) patients in the dual antiplatelet group compared with six (3.8%) patients in the single antiplatelet group (HR, 1.02; 95% CI, 0.94–1.11; *p* = 0.343). Recurrent ischemic events occurred in four (2.5%) patients in the dual antiplatelet group and three (1.9%) patients in the single antiplatelet group (HR, 1.28; 95% CI, 0.99–1.47; *p* = 0.149). Vascular-related death occurred in two (1.3%) patients in the dual antiplatelet group and three (1.9%) patients in the single antiplatelet group (HR, 0.76; 95% CI, 0.51–1.03; *p* = 0.094).

**Table 3:** Outcomes of dual antiplatelet therapy in propensity score-matched cohort.

	Dual antiplatelet (*N* = 157)	Single antiplatelet (*N* = 157)	HR (95%CI)	*p* value
Composite of recurrent ischemic stroke, transient ischemic attack and death (%)				
At 6 months	6 (3.8)	6 (3.8)	1.02 (0.94–1.11)	0.343
At 1 year	10 (6.3)	12 (7.6)	0.74 (0.61–0.90)	0.024
Recurrent ischemic stroke or transient ischemic Attack (%)				
At 6 months	4 (2.5)	3 (1.9)	1.28 (0.99–1.47)	0.149
At 1 year	6 (3.8)	7 (4.5)	0.88 (0.71–1.05)	0.263
Vascular-related death (%)				
At 6 months	2 (1.3)	3 (1.9)	0.76 (0.51–1.03)	0.094
At 1 year	4 (2.5)	5 (3.2)	0.85 (0.69–1.02)	0.118
Number of new ischemic lesions in recurrent ischemic stroke patients (IQR)	3 (2–5)	5 (3–7)	0.88 (0.77–0.98)	0.032
Adverse events (%)				
Intracranial hemorrhage	0	0	–	–
Extracranial hemorrhage	12 (7.6)	9 (5.7)	1.35 (1.13–1.48)	0.017
Major hemorrhage	0	0	–	–
Minor hemorrhage	12 (7.6)	9 (5.7)	1.35 (1.13–1.48)	0.017
Dyspepsia	24 (15.2)	22 (14.0)	1.10 (0.97–1.25)	0.142
Headache	10 (6.4)	8 (5.1)	1.19 (1.00–1.38)	0.102
Dizziness	8 (5.1)	7 (4.5)	1.09 (0.97–1.21)	0.225
Palpitation	7 (4.5)	6 (3.8)	1.16 (0.99–1.30)	0.128

### One year outcomes of dual antiplatelet treatment in propensity score-matched cohort

Table 3 shows the 1-year outcomes of the dual antiplatelet therapy in the propensity score-matched cohort. Composite events occurred in 10 (6.3%) patients in the dual antiplatelet group compared with 12 (7.6%) in the single antiplatelet group (HR, 0.74; 95% CI, 0.61–0.90; *p* = 0.024). Recurrent ischemic events occurred in six (3.8%) patients in the dual antiplatelet group compared with seven (4.5%) in the single antiplatelet group (HR, 0.88; 95% CI, 0.71–1.05; *p* = 0.263). Vascular-related death occurred in four (2.5%) patients in the dual antiplatelet group and five (3.2%) in the single antiplatelet group (HR, 0.85; 95% CI, 0.69–1.02; *p* = 0.118). The number of new ischemic lesions was significantly lower in the dual antiplatelet group (HR, 0.88; 95% CI, 0.77–0.98; *p* = 0.032).

### Adverse events of dual antiplatelet treatment in propensity score-matched cohort

Extracranial hemorrhage occurred in 12 (7.6%) patients in the dual antiplatelet group compared to nine (5.7%) in the single antiplatelet group (HR, 1.35; 95% CI, 1.13–1.48; *p* = 0.017). No intracranial hemorrhages were observed in this cohort.

### Subgroup analysis of composite events and survival analysis of study participants

**Figure 2:**
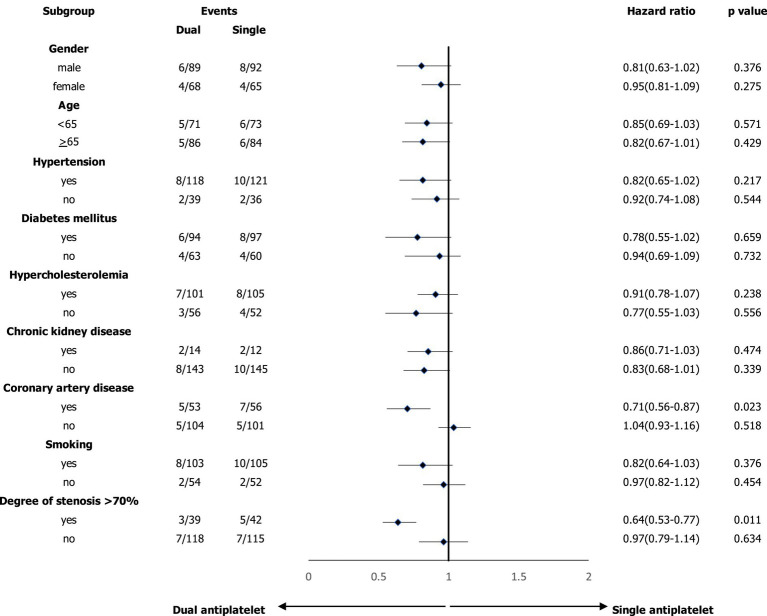
Subgroup analysis based on the composite events.

**Figure 3:**
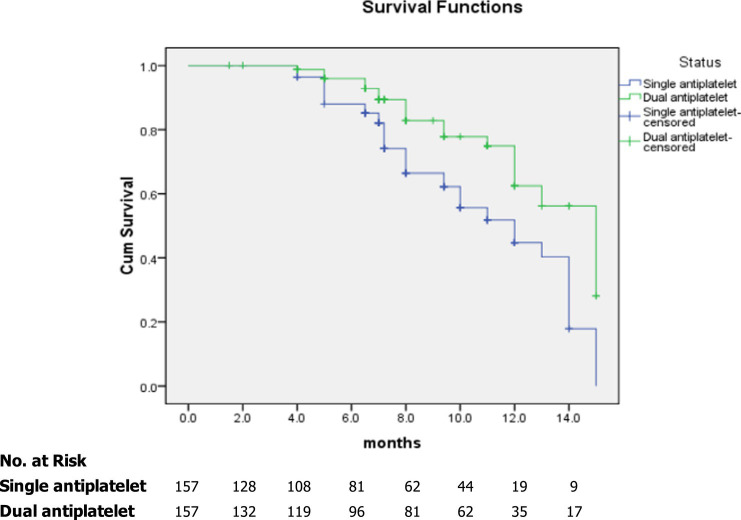
Kaplan–Meier curves showing overall survival among study participants based on composite events.

In the subgroup analyses, we compared the main outcomes between the dual and single antiplatelet groups according to sex, age, hypertension, diabetes mellitus, hypercholesterolemia, chronic kidney disease, coronary artery disease, smoking, and degree of carotid artery stenosis >70% (Figure 2). A reduced risk of composite events was observed in the presence of coronary artery disease, and the degree of stenosis was >70%. Figure 3 shows the survival analysis of the study participants based on the composite events.

## Discussion

In this single-center tertiary care setting, conducted with cilostazol-based dual antiplatelet therapy in patients with asymptomatic atherosclerotic carotid artery stenosis, dual antiplatelet therapy reduced composite events compared with single antiplatelet therapy. The incidence of hemorrhagic complications was higher in patients who received dual antiplatelet therapy for at least 1 year.

The incidence of recurrent cerebral ischemia was 3.8–4.5% in the study participants. However, the method of measuring the degree of carotid artery stenosis using the North American Symptomatic Carotid Endarterectomy Trial (NASCET) (5) or the European Carotid Surgery Trial (ECST) (10) affects the degree of stenosis, which also varies across different studies. A 50% NASCET stenosis equates to a 75% ECST, whereas a 70% NASCET stenosis equates to an 85% ECST (11). The proportion of patients with >70% carotid artery stenosis was approximately 25.5% in this study, potentially explaining why the event rate of ischemic stroke in this study was higher than that reported in other previously published studies (12–14).

This study included multiple vascular risk factors, such as hypertension, diabetes mellitus, hypercholesterolemia, and smoking. Carotid artery stenosis and coronary artery disease may share similar mechanisms owing to atherosclerosis. The presence of platelet-rich atheromatous plaques characterizes atherosclerosis. Therefore, dual antiplatelet therapy can halt atheromatous plaque formation and stabilize unstable atheromatous plaques (15, 16). However, combining it with antiplatelet therapy increased the risk of hemorrhagic complications during long-term clinical follow-up. These results align with those of a recently published study (17, 18).

In this study, all participants with asymptomatic carotid artery stenosis had a recent ischemic stroke or transient ischemic attack in the contralateral carotid territory. Contralateral ischemic stroke is associated with an increased risk of late stroke in patients with medically treated asymptomatic carotid artery stenosis (19). Current treatment approaches for asymptomatic carotid artery stenosis involve medication and lifestyle modifications (20–22). Vascular risk factor control requires time to show results, potentially resulting in a lower incidence of stroke during the follow-up period and supporting the effectiveness of dual antiplatelet therapy. In the dual antiplatelet group, patients with asymptomatic carotid artery stenosis achieved a target blood pressure of 75.4%, HbA1c of 83%, low-density lipoprotein cholesterol level of 75.7%, and smoking cessation rate of 81.1%. The combination of dual antiplatelet therapy and strict vascular risk factor control affected the composite events in this study. Cognitive impairment (23, 24) can indirectly affect patient outcomes, such as medication adherence and lack of stroke awareness. However, the participants in this study had lower Fazekas scale scores and a lower proportion of medication non-adherence.

The treatment of atherosclerosis is based on the use of oral antiplatelet agents. Aspirin is the most commonly used antiplatelet agent for treating asymptomatic carotid artery stenosis. However, evidence of aspirin’s use in asymptomatic carotid artery stenosis is significantly weaker (25, 26). Clopidogrel has not been studied in patients with asymptomatic carotid artery stenosis. Combining antiplatelets with different mechanisms is expected to be more effective than monotherapy in preventing the recurrence of ischemic stroke. Short-term dual antiplatelet treatment with aspirin and clopidogrel prevented recurrent ischemic stroke in acute non-cardioembolic ischemic stroke (27). In this study, 97(56.4%) patients received cilostazol plus clopidogrel; the efficacy of clopidogrel-based dual antiplatelet was shown in many previously published research (27–29). However, most randomized controlled studies have not specifically recruited patients with asymptomatic atherosclerotic carotid artery disease (25, 30, 31). The combination of cilostazol and another antiplatelet agent is expected to decrease stroke recurrence without increasing the risk of hemorrhagic complications. In recent studies (32, 33), cilostazol-based dual antiplatelet therapy is not beneficial in preventing recurrent stroke with extracranial atherosclerosis.

Currently, the optimal medical treatment approach for patients with asymptomatic atherosclerotic carotid artery disease remains unclear. Most primary prevention randomized controlled trials did not specifically recruit asymptomatic patients with carotid artery stenosis (34). Risk stratification is an important aspect of deriving benefits from antiplatelet treatment plans. In subgroup analyses, dual antiplatelet therapy may be beneficial in patients with coronary artery disease or a degree of asymptomatic carotid artery stenosis of >70%. Additionally, atherosclerosis may underlie the pathogenic mechanisms of both carotid artery and small-vessel diseases. Recently, a large prospective observational study (35) demonstrated that dual antiplatelet therapy can improve the outcomes of capsular warning syndrome, which is thought to result from thrombosis of the small arteries affected by atherosclerosis, and is considered the final stage in the progression of lacunar stroke.

This study has several strengths and limitations. The strength of this study is that it directly examined the association between dual antiplatelet efficacy and safety in patients with asymptomatic atherosclerotic carotid artery disease with long-term follow-up. However, this study has some limitations. First, all patients with ischemic stroke and transient ischemic attack received other secondary prevention medications such as antihypertensive, antidiabetic, and lipid-lowering agents. These agents may have affected the efficacy of the antiplatelet agents in the study participants. Second, patients with asymptomatic atherosclerotic carotid artery disease in this study had lower composite outcome rates. The number of outcomes in some subgroups cannot be interpreted as meaningful when divided into subgroups. Third, this study did not consider the potential influence of transient ischemic attack characteristics, which have been shown to be associated with a higher short-term risk of stroke. This is particularly significant given the results of large prospective cohort studies, which have demonstrated a strong association between recurrent ischemic events and a higher risk of stroke (35, 36). Fourth, owing to the retrospective study design and analysis of enrollment data, unmeasured bias or uncollected confounders may have existed. However, this limitation was addressed using a propensity score matching analysis. Finally, the outcomes of this study may need confirmation in a larger study population or randomized controlled study in the future.

## Conclusion

Among patients with asymptomatic carotid artery stenosis, those who received dual antiplatelet therapy had a lower risk of composite events but a higher risk of minor extracranial hemorrhage than those who received single antiplatelet therapy.

## Data availability statement

The raw data supporting the conclusions of this article will be made available by the authors, without undue reservation.

## Ethics statement

The studies involving humans were approved by institutional review board of the Neurological Institute of Thailand. The studies were conducted in accordance with the local legislation and institutional requirements. The participants provided their written informed consent to participate in this study.

## Author contributions

TT: Conceptualization, Data curation, Formal analysis, Methodology, Writing – original draft, Writing – review & editing. CP: Writing – original draft, Writing – review & editing.
